# Oral submucous fibrosis: A clinicopathological study of 674 cases in China

**DOI:** 10.1111/jop.12836

**Published:** 2019-02-18

**Authors:** Xinjia Cai, Zhigang Yao, Gui Liu, Lin Cui, Huiling Li, Junhui Huang

**Affiliations:** ^1^ Department of oral pathology Xiangya Stomatological Hospital Central South University Changsha Hunan China

**Keywords:** areca nut, associated lesions, clinicopathological features, oral submucosa fibrosis

## Abstract

**Background:**

Oral submucous fibrosis (OSF) has been reported frequently in India and other countries in South Asia. There are few reports on the clinicopathological features of OSF in China, where OSF is an epidemic. This study analyses the clinicopathological features of OSF in Hunan Province, China.

**Methods:**

A total of 674 cases of OSF were collected from July 2013 to August 2018 in Xiangya Stomatological Hospital, Central South University, and gender, age, site, pathological stage, habits, symptoms and associated lesions were recorded.

**Results:**

The male to female ratio was 32.7:1. The average age was 35.23 ± 10.08. The buccal mucosa was the most common site. A total of 99.85% of OSF cases chewed areca nut. Pale mucosa, restricted mouth opening, burning and fibrous bands were common clinical manifestations. Oral leukoplakia (OLK) was the most common associated lesion. The extended duration of chewing areca nut increased the risk of associated lesions (*P* < 0.05). The risk of OSF associated with OLK decreased with increasing OSF stage (*P* < 0.05).

**Conclusion:**

The prevalence of OSF in males was higher than that in females, the buccal mucosa was most affected, and chewing areca nut is the most common habit of OSF patients.

## 1 INTRODUCTION

Oral submucous fibrosis (OSF) is a chronic progressive inflammatory disease that primarily affects the oral mucosal epithelium and lamina propria. In previous studies, OSF patients often chewed areca nut with epithelial atrophy, and fibrosis in the lamina propria caused restricted mouth opening.^1^, ^2^ The prevalence of OSF has increased from 8.3/10^5^ to 16.2/10^5^ in recent years.^3^ OSF used to be reported only in Southeast Asia, but it is now found in the Asian immigrant populations of Britain and America and has become a worldwide health problem.^4^ OSF is an oral potential malignant disorder (OPMD).^5^ The malignant rate of OSF is 9.13%, and the risk of cancer in OSF patients is 29.26 times that of non‐OSF patients.^6^, ^7^ OSF has been reported frequently in India and other countries in South Asia. There are few reports on the clinicopathological features of OSF in China, where OSF is an epidemic. To investigate OSF further, it is necessary to analyse features of patients in China. The clinical management of OSF is important for the prevention of oral cancer. This study analyses the clinicopathological features of OSF in Hunan Province, China, for the prevention and early diagnosis of the disease.

## 2 MATERIALS AND METHODS

A total of 674 cases of OSF were diagnosed by two experienced pathologists at the Department of Oral Pathology, Xiangya Stomatological Hospital, Central South University, from July 2013 to August 2018 (5‐year period). Criteria for the clinical diagnosis of OSF were the presence of pale mucosa, fibrous bands, burning sensation or restricted mouth opening. Histopathological changes of OSF observed were fibrous degeneration of connective tissue, mild hyalinization of collagen fibrous tissue beneath the epithelium (early stage), moderate hyalinization of collagen fibrous tissue beneath the epithelium with mild oedema (middle stage), and hyalinization of total collagen fibrous beneath the epithelium with stenosis or occlusion of vessels (advanced stage).^8^ All cases were diagnosed by incisional biopsy. All cases included a complete history, such as gender, age, site, pathological stage, chewing habits, symptoms and associated lesions. This study was approved by the medical ethics committee of Xiangya Stomatological Hospital, Central South University.

### 2.1 Statistical analysis

Gender, age, site, pathological stage, habits, symptoms and associated lesions of OSF were collected. All statistical analyses were performed using SPSS 24.0 software. Chi‐square tests were performed for comparing percentages of two independent samples and the differences between China and India (Table 1). A one‐way ANOVA test was performed on the ages at different pathological stages. *P* < 0.05 was considered statistically significant.

**Table 1:** Differences of clinicopathological features of OSF between China and India

Variable	China (%)	India (%)^12^	*P* value
Total	674	1000	
Gender			
Male	654 (97.03)	830 (83.00)	0.000
Female	20 (2.97)	170 (17.00)	
Age			
0‐9	0 (0.00)	1 (0.10)	0.000
10‐19	16 (2.37)	203 (20.30)	
20‐29	219 (32.49)	481 (48.10)	
30‐39	227 (33.68)	190 (19.00)	
40‐49	144 (21.36)	85 (8.50)	
>50	68 (10.09)	40 (4.00)	
Habits			
Chewing areca nut	673 (99.85)	594 (59.40)	0.000
Smoking tobacco	176 (26.11)	324 (32.40)	0.006
Symptoms			
Restricted mouth opening	382 (56.68)	908 (90.80)	0.000
Burning	350 (51.93)	891 (89.10)	0.000
Associated lesions			
OLK	101 (14.99)	48 (4.80)	0.000
OLP	2 (0.30)	7 (0.70)	0.268

OLK, oral leukoplakia; OLP, oral lichen planus; OSF, oral submucous fibrosis.

## 3 RESULTS

A total of 674 cases of OSF were collected. The male to female ratio was 32.7:1. The age range was 16‐67 years, and the mean age was 35.23 ± 10.08 years old. OSF was most frequent in the age range of 30‐39 years (33.68%). The main site of OSF was the buccal mucosa (88.87%), followed by the tongue (7.57%), lip (1.93%), palate (1.34%) and gingiva (0.30%). Of the OSF cases, 99.85% had chewed areca nut, with 23.74% smoking tobacco. The OSF patients presented with pale mucosa, restricted mouth opening, burning and fibrous bands.

### 3.1 Pathological stages

**Figure 1:**
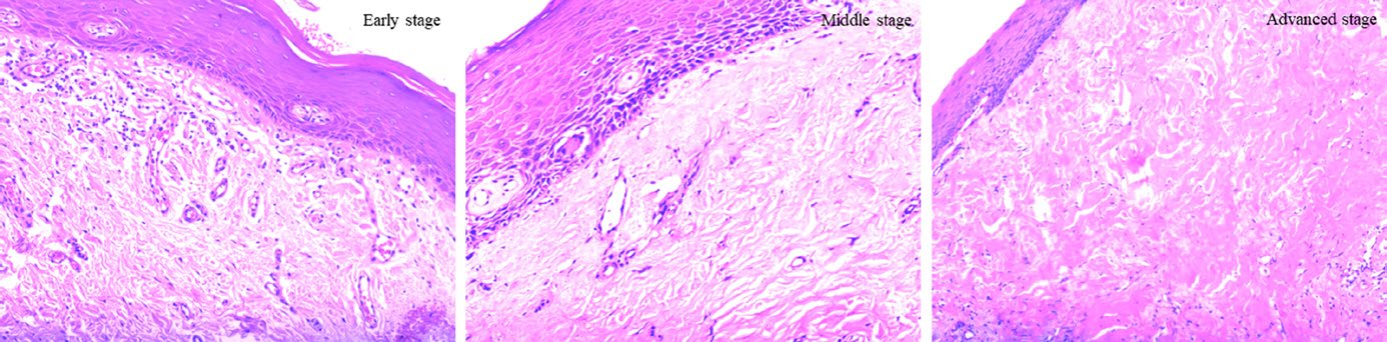
OSF (×200). Early stage: Fibrous degeneration of connective tissue, mild hyalinization of collagen fibres beneath the epithelium. Middle stage: Fibrous degeneration of connective tissue, moderate hyalinization of collagen fibres beneath the epithelium with mild oedema. Advanced stage: Fibrous degeneration of connective tissue, hyalinization of total collagen fibres beneath the epithelium with stenosis of vessels

Oral submucous fibrosis was most frequently observed in the middle stage (76.11%; Figure 1). The mean ages of various stages of OSF were 39.69 ± 9.66 (early), 35.16 ± 10.12 (middle) and 31.18 ± 8.14 (advanced), and the age decreased with increasing stage (*P* < 0.05). One hundred and sixty patients who chewed areca nut with smoking tobacco did not have an increased risk of pathologic stage of OSF compared to patients chewing areca nut only (*P* > 0.05). No significant difference in the duration of chewing areca nut and OSF stage was observed (*P* > 0.05).

### 3.2 Associated lesions

**Figure 2:**
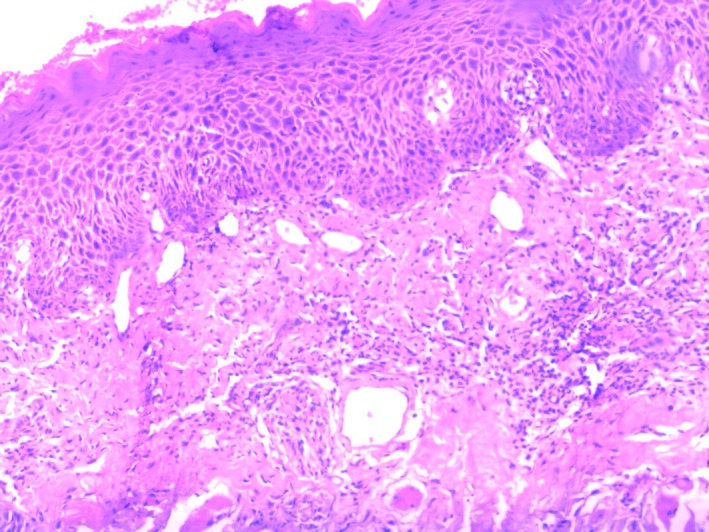
OSF associated with OLK (×200). Fibrous degeneration of connective tissue, mild epithelial dysplasia, disappearance of basal cell polarity, epithelial rete peg hypertrophy

**Figure 3:**
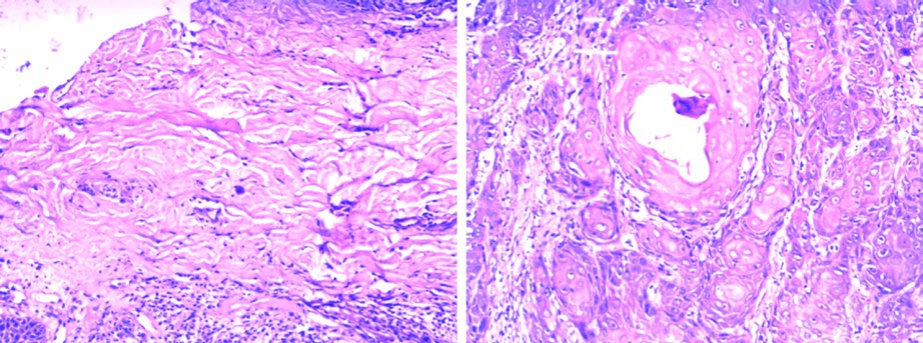
OSF associated with well‐differentiated OSCC (×200). Fibrous degeneration of connective tissue; proliferating epithelium invades the connective tissue, forming the keratin pearl

**Figure 4:**
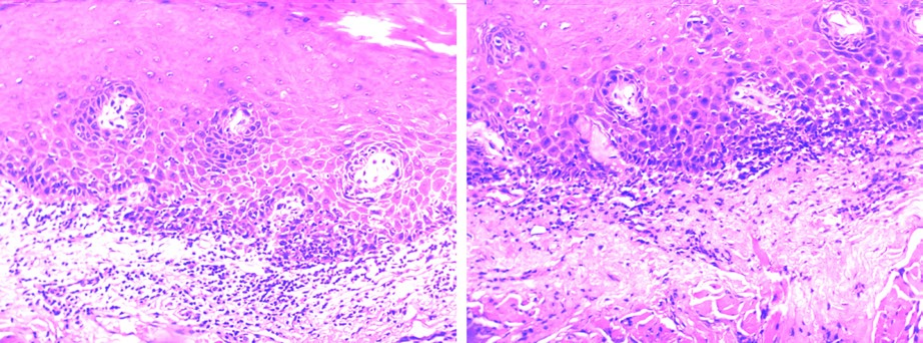
OSF associated with OLP (×200). Fibrous degeneration of connective tissue, liquefaction degeneration of the basal layer, lymphocyte infiltration at mucosal lamina propria

Several associated lesions were oral leukoplakia (OLK) (14.99%), oral squamous cell carcinoma (OSCC) (0.74%) and oral lichen planus (OLP) (0.30%; Figures 2, 3, 4). All cases of OSCC found were well differentiated. Patients chewing areca nut with smoking tobacco did not have an increased risk of associated lesions (*P* > 0.05). Extended duration (>10 years) of chewing betel nut did increase the risk of associated lesions of OSF, such as OLK, OSCC and OLP (*P* < 0.05). With increasing OSF stage, OSF associated with OLK significantly decreased (*P* < 0.05).

## 4 DISCUSSION

Oral submucous fibrosis was first proposed as a precancerous condition by Pindborg, who found a malignancy rate of 7.6%.^9^, ^10^ OSF was mainly reported in Asia, especially in India and southern China, with other reports from Pakistan, Sri Lanka, Bangladesh, Malaysia, Singapore, Thailand and Saudi Arabia. OSF was also observed in the Asian populations of Britain and America and is considered a worldwide health problem.^11^

The differences in the clinicopathological features of OSF in this study compared to those in another detailed study are listed in Table 1. The proportion of males in China was significantly higher than that in India. Studies in Taiwan and India also showed more males with OSF, and the male to female ratio is 4.9:1~11:1.^3^, ^12^, ^13^, ^14^ All male‐female ratios are lower in this study. This may be related to the fact that non‐Chinese females usually chewed areca nut and tobacco, while Chinese females rarely chewed it. OSF patients in China were older than patients in India. However, studies showed that the mean age of OSF was 46 (India) and 39 (Pakistan), which are both higher than the 35 years found in this study.^14^, ^15^ A total of 99.85% of OSF patients chewed areca nut in China, while 59.40% chewed areca nut in India. However, more patients smoked tobacco in India than in China. The symptoms of restricted mouth opening and burning of Indian patients were more severe than in this study. This might be associated with the chewing habits of patients. The patients in India chewing areca nut, kharra, gutkha, tobacco, snuff and betel quid were different from the patients from this study.^12^

Almost every patient with OSF in China chewed areca nut. Areca nut is different from the foreign chewing tobacco, such as kharra and gutkha. Only 79% of Indian patients with OSF chewed areca nut, which was lower than the 99.85% of Chinese patients in this study.^14^ A study in Hunan, China showed that all OSF patients chewed betel nut, and the proportion of male was significantly higher than female, which was similar to this study.^16^ Tilakaratne et al found a relationship between chewing areca nut and OSF in India, Pakistan, Sri Lanka, Taiwan, and immigrants of South Africa and Britain.^17^ The areca nut extract induced contraction of buccal mucosal fibroblasts, indicating that they were involved in the pathogenesis of OSF.^18^ Sarode et al reported that areca nut did not cause OSF. Areca nut mixed with slaked lime led to more transformation of normal fibroblasts. Fibroblasts stimulated by arecoline resulted in collagen fibre formation, which caused OSF. Another important role of slaked lime is promoting the penetration of arecoline into the inflammatory oral mucosa.^19^ This study reported the association between OSF stage and chewing habits. The extension of the duration of chewing areca nut and smoking tobacco did not increase the risk of advanced OSF (*P* > 0.05). The primary pathological manifestation of OSF was fibrosis of the connective tissue. The pathological stage of OSF depends on the degree of collagen fibre glassiness, oedema and lymphocyte infiltration.^8^ Smoking did not increase the degree of connective tissue degeneration in OSF. Extending the duration of chewing areca nut did not increase the risk of OSF. The increase in OSF stage may be related to the frequency and number of areca nuts. Ali et al found that patients affected by early OSF were more correlated with gutkha and areca nut (*P* < 0.05). The duration and frequency of chewing gutkha or areca nut with smoking were not statistically related to OSF.^20^ Ara et al identified that chewing gutkha was the major risk factor for OSF, especially in young age groups. As the duration and frequency increased, the severity of OSF increased, but the clinical stage was not related to pathological grade.^21^

The youngest OSF patient was 16 in this study. Agrawal et al reported a case of OSF in a 9‐year‐old Indian girl with a history of chewing areca nut.^22^ Dhariwal et al demonstrated 2 cases of Indian OSF in children, specifically, a 10‐year‐old boy with a history of chewing gutkha and a 12‐year‐old girl with a history of chewing pan masala.^23^ Shah et al reported an 11‐year‐old Bangladeshi girl with OSF who had a history of chewing areca nut and supari.^24^ Chaturvedi et al found that patients with oral cancer caused by OSF were younger than non‐OSF patients,^25^ suggesting that children chewing areca nut could suffer from OSF. Parents and children were not aware of the harm of areca nut. The mean ages of OSF in the early (81 cases), middle (513 cases) and advanced (80 cases) stages were 39.69 ± 9.66, 35.16 ± 10.12 and 31.18 ± 8.14, respectively, which decreased with increasing stage (*P* < 0.05), suggesting that young patients chewing areca nut might lead to an advanced stage of OSF. This may be related to the high susceptibility in a small population. More studies need to be performed to illustrate the specific mechanism. To prevent OSF, it is important to strengthen the management of youth to stop them from chewing areca nut.

The lesion most associated with OSF was OLK (14.99%), which was higher than the 4.8% found in India.^12^ Following OSCC and OLP, no erythema was found. All OSCCs with OSF were well differentiated. Although studies have shown that smoking increases the risk of OLK and OSCC,^26^, ^27^ in this study, smoking tobacco did not significantly increase the risk of OSF associated with OLK and OSCC (*P* > 0.05). This suggests that risk factors such as smoking tobacco play complicated roles in associated lesions. Few studies have reported on the pathogenesis of associated lesions, and more detailed studies are needed. As the duration of chewing areca nut is extended, the risk of OSF with associated lesions increased (*P* < 0.05). Oral diseases such as OSF, OLK, OLP and OSCC were related to chewing areca nut, so the risk of OSF associated with OLK, OLP and OSCC increased with the extended duration of chewing areca nut.^28^ The risk of OSF associated with OLK decreased with the increase in pathological stage (*P* < 0.05). Early‐stage OSF with a low degree of atrophied epithelium, fibrosis tissue and collagen fibre glassiness was more likely to cause epithelial hyperplasia and dysplasia associated with OLK. OSF might be caused by the epithelial‐mesenchymal transition.^29^ The transition of epithelial cells to a mesenchymal phenotype might affect epithelial hyperplasia and decrease the occurrence of OLK. In addition, the fibrosis of sub‐epithelial connective tissue was expected to affect the epithelial components.^30^ The incidence of OLK decreases with the severity of fibrosis. The specific mechanism still needs more research in the future. The malignant transformation cannot be identified from the current cases of OSF. There was no information about nutritional status. The case of malignant transformation from OSF and nutritional status should be collected and analysed in the future.

## 5 CONCLUSION

This study was performed to analyse the clinicopathological features of OSF in China. Males were preponderant, and the buccal mucosa was most affected. Almost all patients chewed areca nut. Interesting findings were the increased risk of OSF‐associated lesions with extended duration of chewing betel nut. A minority of individual appeared to experience an advances stage of disease at a younger mean age suggesting high susceptibility in a small population. Education regarding the risks of the use of betel quid and the development of OSF should include younger individuals and parents.

## CONFLICT OF INTEREST

All authors agreed that there are no competing interests.
